# Another character theoretic formula for base size

**DOI:** 10.1007/s00013-025-02165-3

**Published:** 2025-08-19

**Authors:** Coen del Valle

**Affiliations:** School of Mathematics and Statistics, The Open University, Milton Keynes, UK

**Keywords:** Base size, Irreducible character, Külshammer graph, 20B05, 20B30

## Abstract

A base for a permutation group *G* acting on a set $\Omega$ is a sequence $\mathcal {B}$ of points of $\Omega$ such that the pointwise stabiliser $G_{\mathcal {B}}$ is trivial. The base size of *G* is the size of a smallest base for *G*. Extending the results of a recent paper of the author, we prove a 2013 conjecture of Fritzsche, Külshammer, and Reiche. Moreover, we generalise this conjecture and derive an alternative character theoretic formula for the base size of a certain class of permutation groups. As a consequence of our work, a third formula for the base size of the symmetric group of degree *n* acting on the subsets of $\{1,2,\dots , n\}$ is obtained.

## Introduction

A *base* for a permutation group *G* acting on a finite set $\Omega$ is a sequence $\mathcal {B}$ of points of $\Omega$ with trivial pointwise stabiliser $G_\mathcal {B}$. The size *b*(*G*) of a smallest base for *G* is called the *base size* of *G*. In 1992, Blaha  [1] showed that the problem of finding a minimum base for an arbitrary group *G* is NP-hard. Despite this, much work has been done towards determining the base size of certain families of groups, especially primitive groups; we recommend the paper of Maróti [8] for a survey of many significant results in the area.

Let *n* and *k* be positive integers with $n\ge 2k$ and let $\textrm{S}_{n,k}$ be the symmetric group $\textrm{S}_n$ acting on the *k*-element subsets of $\{1,2,\dots ,n\}$. The group $\textrm{S}_{n,k}$ is primitive provided that $n>2k$. In 2013, the base size of $\textrm{S}_{n,k}$ was determined by Fritzsche, Külshammer, and Reiche [7, Lemma 3.3] in a beautiful result which appears to have either gone unnoticed, or forgotten in the literature—one purpose of this paper is to rectify this oversight. Over 10 years later, two independent papers [6, 9] were published, once again determining formulae for $b(\textrm{S}_{n,k})$; the formula in [6, Theorem 1.1] is the same as that which appears in [7], but the formula of Mecenero and Spiga [9, Theorem 1.1] takes a remarkably different form. During the review process, it was noted that the result [9, Theorem 1.1] has an entirely character theoretic interpretation. In particular, after some straightforward algebraic manipulation, one derives that if $\textrm{sgn}$ is the sign character of $\textrm{S}_n$ and $\chi$ is the permutation character of $\textrm{S}_{n,k}$, then

$$b(\text {S}_{n,k})=\min \{l\in \mathbb {N}: \langle \chi ^l, \text {sgn}\rangle \ne 0\}.$$

A recent paper of the author [5] exhibited an entirely algebraic proof of the above formula, and extended it to all groups admitting a *base-controlling* homomorphism. We say that ${\phi :G\rightarrow \{1,-1\}}$ is *base-controlling* if for every tuple $\mathcal {A}$ of points of $\Omega$, $\mathcal {A}$ is a base if and only if $\phi (G_{\mathcal {A}})=1$. Note that any base-controlling homomorphism of *G* is an irreducible character of *G*. Define $\langle -, - \rangle$ to be the standard inner product of (complex-valued) class functions. That is, for class functions $\varphi _1,\varphi _2$ of *G*,

$$\langle \varphi _1,\varphi _2\rangle =|G|^{-1}\sum _{g\in G}\varphi _1(g)\overline{\varphi _2(g)}.$$

One main result of [5, Theorem 1.2] is that if $\chi$ is the permutation character of a group *G* which admits a base-controlling homomorphism $\phi$, then

1 $$\begin{aligned} b(G)=\min \{l\in \mathbb {N} :\langle \chi ^l, \phi \rangle \ne 0\}. \end{aligned}$$

The paper [7] concludes with a lovely conjecture, suggesting an alternative character theoretic formula for the base size of $\textrm{S}_{n,k}$. Before stating their conjecture, we first give the necessary background.

Let *G* be a finite transitive permutation group with point stabiliser *H*. Define the *Külshammer graph* $\mathcal {K}(G,H)$ to have vertex set $\textrm{Irr}(H)$ where $\alpha$ is adjacent to $\beta$ if and only if the induced characters $\alpha {\uparrow }^G$ and $\beta {\uparrow }^G$ have a common irreducible constituent. By [3, Corollary 6.3], the graph $\mathcal {K}(G,H)$ is connected; for any $\alpha ,\beta \in \textrm{Irr}(H)$, define $d(\alpha ,\beta )$ to be the length of a shortest path from $\alpha$ to $\beta$ in $\mathcal {K}(G,H)$. Define the *diameter* of $\mathcal {K}(G,H)$ to be $\textrm{Diam}(\mathcal {K}(G,H))=\max _{\alpha ,\beta \in \textrm{Irr}(H)}d(\alpha ,\beta ).$

The Külshammer graph has been studied several times in the past (see e.g. [3, 4, 7]), primarily for its utility in determining the *depth* of a subgroup *H* in a group *G*. The definition of this notion of depth is beyond the scope of this paper but we point the interested reader to [2–4, 7] for further discussion and to [4, Proposition 2.5] for a nice collection of facts relating the combinatorial information of the Külshammer graph to depth.

We are now ready to present the conjecture of Fritzsche, Külshammer, and Reiche [7]; throughout this paper, we use $1_H$ to denote the trivial character of a group *H*, and use $\hspace{0.07cm}\hspace{-0.07cm}\uparrow ^G$ and $\hspace{0.07cm}\hspace{-0.07cm}\downarrow _H$ to denote the induction and restriction of characters, respectively.

### Conjecture 1.1

([7]). Let $n\ge 2k$, let $G=\textrm{S}_{n,k}$, and let *H* be a point stabiliser of *G*. Then

$$b(G)=\textrm{Diam}(\mathcal {K}(G,H))+1=d(1_H,\textrm{sgn}\hspace{-0.07cm}\downarrow _H)+1.$$

In this paper, we settle Conjecture 1.1; our main result is the following, which gives us another character theoretic formula for base size, distinct from that of [5].

### Theorem 1.2

Let *G* be a finite transitive permutation group with point stabiliser *H*. Suppose that *G* admits a base-controlling homomorphism $\phi$. Then

$$b(G)=\textrm{Diam}(\mathcal {K}(G,H))+1=d(1_H,\phi \hspace{-0.07cm}\downarrow _H)+1.$$

Since the sign character is base-controlling for $G=\textrm{S}_{n,k}$ (see [5, Sect. 3] for details), we immediately deduce the following.

### Corollary 1.3

The Fritzsche–Külshammer–Reiche conjecture is true.

Thus, we obtain a third formula for the base size of $\textrm{S}_{n,k}$.

The structure of this paper is straightforward. In Section 2, we present a proof of Theorem 1.2, and in Section 3, we work through a couple of easy examples. Throughout, we assume familiarity with some basic concepts in character theory, and refer the reader to [10] for the necessary background.

## Proof of Theorem 1.2

Throughout this section, *G* is a finite transitive permutation group with base-controlling homomorphism $\phi$ and point stabiliser *H*, and $\chi =1_H\hspace{-0.07cm}\uparrow ^G$ is the permutation character of *G*. Before stating our key proposition, we remind the reader of a standard result of character theory which will be very useful for us: if $\alpha$ is any character of *G*, then

2 $$\begin{aligned} \alpha \hspace{-0.07cm}\downarrow _H\hspace{-0.07cm}\uparrow ^G=\alpha \cdot \chi . \end{aligned}$$

The equality (2) is a special case of [10, Chapter 7.2, Remark (3)].

### Proposition 2.1

Let $1_H,\alpha _1,\alpha _2,\dots ,\alpha _m$ be a path in $\mathcal {K}(G,H)$. Then

$$\begin{aligned} \langle \chi ^k,\alpha _k{\uparrow }^G\rangle \ne 0 \end{aligned}$$

for all $$1\le k\le m$$.

### Proof

We prove the result by induction. The result holds for $k=1$: Indeed, since $1_H$ and $\alpha _1$ are joined by an edge, it follows that $0\ne \langle 1_H\hspace{-0.07cm}\uparrow ^G,{\alpha _1\hspace{-0.07cm}\uparrow ^G}\rangle =\langle \chi ^1,\alpha _1\hspace{-0.07cm}\uparrow ^G\rangle ,$ as desired. Assume the result holds for some $k\ge 1$. Then

$$0\ne \langle \chi ^k,\alpha _k\hspace{-0.07cm}\uparrow ^G\rangle =\langle \chi ^k\hspace{-0.07cm}\downarrow _H,\alpha _k\rangle $$

by Frobenius reciprocity. That is, $\alpha _k$ is an irreducible constituent of $\chi ^k\hspace{-0.07cm}\downarrow _H$. Additionally, there is an edge between $\alpha _k$ and $\alpha _{k+1}$, whence

$$0\ne \langle \alpha _k\hspace{-0.07cm}\uparrow ^G,\alpha _{k+1}\hspace{-0.07cm}\uparrow ^G\rangle =\langle \alpha _k,\alpha _{k+1}\hspace{-0.07cm}\uparrow ^G\hspace{-0.07cm}\downarrow _H\rangle .$$

It follows that $\alpha _k$ is a common irreducible constituent of both $\alpha _{k+1}\hspace{-0.07cm}\uparrow ^G\hspace{-0.07cm}\downarrow _H$ and $\chi ^k\hspace{-0.07cm}\downarrow _H$, thus

$$0\ne \langle \chi ^k\hspace{-0.07cm}\downarrow _H,\alpha _{k+1}\hspace{-0.07cm}\uparrow ^G\hspace{-0.07cm}\downarrow _H\rangle =\langle \chi ^k\hspace{-0.07cm}\downarrow _H\hspace{-0.07cm}\uparrow ^G,\alpha _{k+1}\hspace{-0.07cm}\uparrow ^G\rangle =\langle \chi ^{k+1},\alpha _{k+1}\hspace{-0.07cm}\uparrow ^G\rangle ,$$

where the final equality is (2), hence the result. $\square$

We are now ready to prove Theorem 1.2.

### Proof of Theorem 1.2

In [7, Corollary 1.4], it is shown that

$$b(G)\ge \textrm{Diam}(\mathcal {K}(G,H))+1,$$

and moreover, it is clear that $d:=d(1_H,\phi \hspace{-0.07cm}\downarrow _H)\le \textrm{Diam}(\mathcal {K}(G,H)),$ so it suffices to show that $b(G)\le d+1$.

Since $\phi$ is linear, it follows that $\phi \hspace{-0.07cm}\downarrow _H\in \textrm{Irr}(H)$. Thus, by Proposition 2.1,

$$0\ne \langle \chi ^{d},\phi \hspace{-0.07cm}\downarrow _H\hspace{-0.07cm}\uparrow ^G\rangle =\langle \chi ^{d}\hspace{-0.07cm}\downarrow _H,\phi \hspace{-0.07cm}\downarrow _H\rangle =\langle \chi ^{d}\hspace{-0.07cm}\downarrow _H\hspace{-0.07cm}\uparrow ^G,\phi \rangle =\langle \chi ^{d+1},\phi \rangle $$

by repeated applications of Frobenius reciprocity and (2). Finally, since $\langle \chi ^{d+1},\phi \rangle \ne 0$, we deduce from (1) that $b(G)\le d+1$, as was to be shown. $\square$

## Other examples

In this section, we present a couple of easy worked examples, demonstrating the utility of Theorem 1.2 beyond the groups $\textrm{S}_{n,k}$.

**Fig. 1:**
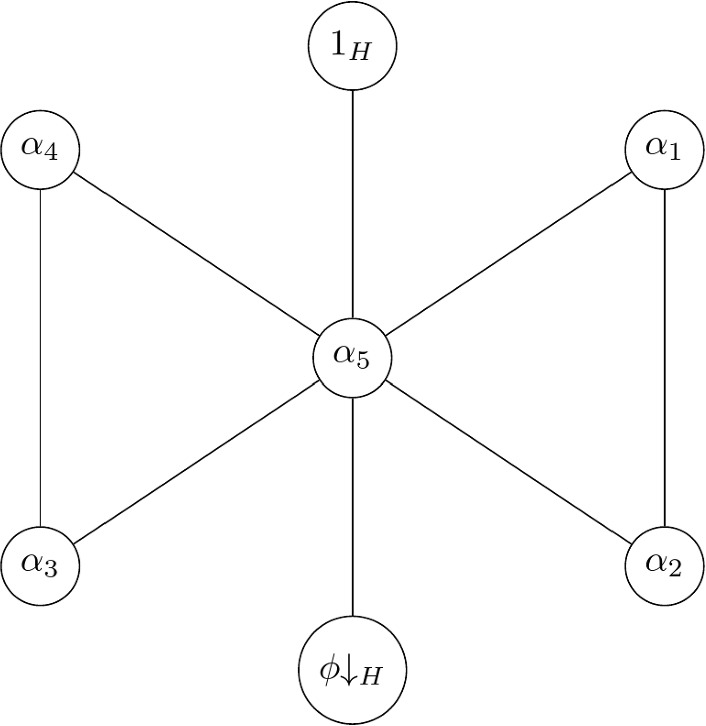
The graph $$\mathcal {K}(\textrm{PGL}_2(7),7\mathord {{\!\,{:}\,\!}}6)$$

First we let $G=\textrm{PGL}_2(7)$ with its natural action on the 1-dimensional subspaces of the natural module $\textrm{GF}(7)^2$. Then *G* has point stabiliser $H=7\mathord {{\!\,{:}\,\!}}6$, and *G* admits a base controlling homomorphism $\phi$ (see [5, Sect. 3] for details). The stabiliser *H* has seven irreducibles characters which we label as $1_H,\phi \hspace{-0.07cm}\downarrow _H,\alpha _1,\alpha _2,\alpha _3,\alpha _4,\alpha _5$. Consider the graph $\mathcal {K}(G,H)$, which is depicted in Fig. 1. It is clear that the graph has diameter 2, and moreover, the distance between $1_H$ and $\phi \hspace{-0.07cm}\downarrow _H$ is indeed 2. This agrees with what we expect since *G* is sharply 3-transitive and thus has base size 3.

We conclude the paper with a final example. Let $G=\textrm{D}_{2n}$ be a dihedral group of degree $n\ge 2$ equipped with its natural action and point stabiliser *H*. Then *H* has exactly two irreducible characters, and it is straightforward to check that the unique non-trivial character is the restriction to *H* of a base-controlling homomorphism $\phi$ of *G*. Since $\mathcal {K}(G,H)$ is necessarily connected, we deduce that $\textrm{Diam}(\mathcal {K}(G,H))+1=d(1_H,\phi \hspace{-0.07cm}\downarrow _H)+1=2$, which agrees with the expected base size.
